# Factors associated with self-care practice among adult diabetes patients in West Shoa Zone, Oromia Regional State, Ethiopia

**DOI:** 10.1186/s12913-018-3448-4

**Published:** 2018-09-24

**Authors:** Yonas Gurmu, Debela Gela, Fekadu Aga

**Affiliations:** 1 Department of Nursing, College of Medicine & Health Sciences, Ambo University, P. O. Box: 19, Ambo, Ethiopia; 2 Department of Nursing & Midwifery, School of Allied Health Sciences, College of Health Science, Addis Ababa University, P.O. Box: 4412, Addis Ababa, Ethiopia; 3 Department of Nursing & Midwifery, School of Allied Health Sciences, College of Health Science, Addis Ababa University, P.O. Box: 9083, Addis Ababa, Ethiopia

**Keywords:** Self-care, Diabetes, Social support, Diabetes knowledge, Self-efficacy, Diabetes duration

## Abstract

**Background:**

Diabetes, a rising global health problem, requires continuous self-care practice to prevent acute and chronic complications. However, studies show that few diabetes patients practice the recommended self-care in Ethiopia. The aim of this study was to assess factors associated with self-care practice among adult diabetes patients in public hospitals of West Shoa Zone, Oromia Regional State, Ethiopia.

**Methods:**

In this cross-sectional study, 257 diabetes patients (mean age 42.9 ± 14.6 years, 54.1% male) completed the survey in Afan Oromo and Amharic languages. A questionnaire consisting standardized tools was used to collect the data. Descriptive and logistic regression analyses were conducted using SPSS version 21.

**Results:**

The mean score for diabetes self-care was 39.8 ± 9.5 and 45.5% of the participants scored below the mean. Multiple logistic regression analysis revealed that having higher diabetes knowledge (AOR = 2.42, 95% CI = 1.22, 4.80), self-efficacy (AOR = 3.30, 95% CI = 1.64, 6.62), social support (AOR = 2.86, 95% CI = 1.37, 5.96), secondary school education (AOR = 6.0, 95% CI = 1.90, 18.85), and longer duration of diabetes (AOR = 5.55, 95% CI = 2.29, 13.44) were important predictors of good diabetes self-care practice.

**Conclusion:**

The diabetes education programs should use strategies that enhance patients’ diabetes knowledge, self-efficacy, and social support. Patients with recent diabetes diagnosis need special attention as they may relatively lack knowledge and skills in self-care. Further studies are needed to elucidate pathways through which diabetes knowledge, self-efficacy, social support, and health literacy affect diabetes self-care.

## Background

Diabetes is one of the major global health problems [1] that requires continuous self-care practices for the prevention of both acute and long-term complications [2]. About 415 million of the world population between 20 to 79 years old lives with diabetes in 2015 alone and approximately 75% of these live in low-and middle-income countries [1]. The global age-standardized diabetes prevalence is 9.0% in men and 7.9% in women. [3] The number of people living with diabetes will likely double in the coming decades [1, 4, 5]. It will even more than double in sub-Saharan Africa where the rapid demographic, sociocultural, and economic transitions are increasing the risk and prevalence of diabetes [6–9].

In the face of scarce resources and a weak healthcare system, Ethiopia – a sub-Saharan country – has approximately 1.3 million adult DM population [1, 10]. Though there is no reliable national prevalence estimate for diabetes in Ethiopia, a community-based study from the northern part of the country reported 5.1% for urban dwelling adults above 35 years old and 2.1% for rural dwelling counterparts [11]. However, other studies show a prevalence of 7% among government employees in the eastern part of the country, [12] 8% among HIV/AIDS patients on antiretroviral therapy in the south, [13] and 6.5% among teachers and Bank employees in Addis Ababa [14]. Diabetes also constitutes 6.5% of the causes for hospital admission in Ethiopia, with diabetic foot ulcer/gangrene and cardiovascular disease being the main indications for admission [15].

Self-care practices are important for the prevention of complications and improved health outcomes in diabetes patient. Self-care practices in diabetes involve healthy eating, medication adherence, blood glucose monitoring, being physically active, and healthy coping [16]. Optimum self-care practice in diabetes is associated with improved glycemic control, [17–19] prevention of cardiovascular risk factors, [19–21] and decrease in unnecessary healthcare utilization, [22] and improved diabetes specific quality of life [23]. Nevertheless, many patients face challenges in implementing the recommended diabetes self-care practices. The challenges may emanate from the various personal and environmental contexts in which self-care practices take place. Some of these determinant factors are personal belief in treatment effectiveness, diabetes knowledge, self-efficacy, self-care skills, social support, and patient-provider communication [24–26]. More specifically, studies show that diabetes duration less than 10 years, male gender, older age, low income being employed, missed diabetes education in the last year, [27] lower self-efficacy or self-care confidence, [27, 28] and higher psychological distress and lower social support [28] are determinants of poor diabetes self-care practice.

The few available studies in Ethiopia show that not many diabetes patients practice the recommended self-care practice [29–31]. According to these studies, more than 60% of diabetes patients had poor diabetes self-care practice. Nevertheless, we have little information concerning factors that could influence the diabetes patient’s engagement in self-care practice in this country. Therefore, this study attempted to fill the knowledge gap in the area by exploring factors associated with diabetes self-care practices. Understanding these factors is important in a multicultural society like Ethiopia since different personal and environmental contexts including demographic, psychosocial, and socioeconomic variables could influence self-care practice in diabetes [32, 33]. The purpose of this study was to assess the demographic, clinical, and psychosocial variables associated with self-care practice among adult diabetes patients on follow up care in public hospitals of the West Shoa Zone, Oromia Regional State, Ethiopia.

### Theoretical framework

This study was informed by the Theory of Diabetes Self-Care Management (TDSCM) [34] that was built using concepts derived from Orem’s self-care theory [35] and Bandura’s self-efficacy theory [36]. The TDSCM claims that personal and environmental factors affect the individual’s engagement in self-care practice to achieve the desired outcome. The personal factors involve gender, age, marital status, religion, education, diabetes knowledge, and self-efficacy while the environmental factors mainly refer to social support. The TDSCM was selected as an organized framework in this study because of its cultural relevance and appropriateness to analyze the association of aforementioned personal and environmental variables with diabetes self-care practice.

## Methods

### Study design, setting, and population

This cross-sectional study was conducted from March–April 2017 in four public hospitals located in the West Shoa zone: Ambo General Hospital, Guder Hospital, Gindeberet Hospital, and Gedo Hospital. All adult (18 years or older) diabetes patients on follow care were included except those who were critically ill at the time of data collection. The net sample of 257 was recruited using a disproportionate stratified sampling method to ensure representativeness between hospitals since they had greatly unequal diabetes patient population. Accordingly, we have selected 133 patients from Ambo General Hospital, 41 from Guder Hospital, 56 from Gedo Hospital, and 27 from Gindeberet Hospital. The data was collected in two local languages – Afaan Oromo and Amharic languages using interviewer-administered questionnaire. Four nurses holding Bachelor of Science degree were involved in data collection under the supervision of a senior nurse professional. The principal investigator also made continues follow up in order to ensure the quality of the data collected. The research project was reviewed by an Institutional Review Board of the College of Health Sciences at Addis Ababa University. Permission to conduct the research was obtained from the authorities in the study settings and written informed consents were secured from each participant.

### Measurements

Before data collection, we took measures to ensure meaning equivalence between the original English version of the questionnaire and the versions in the two local languages. In this regard, the questionnaire was translated from English to Afaan Oromo and Amharic languages by a multilingual translator and then back translated to English by another multilingual translator.

Demographic characteristics of the participants were recorded using 6-items and clinical characteristics with 4-items. The *Summary of Diabetes Self-Care Activities (SDSCA)* [37] was used to measure five areas or domains of diabetic self practices: general diet, specific diet, exercise, medication and self-monitoring of blood glucose. The SDSCA measures the frequency of performing diabetes self-care activities in the last 7 days and had high average inter-item correlations within scales (mean = 0.47) in previous study [37]. A previous psychometric study [38] has shown that SDSCA had a content validity index of 0.83 and a Cronbach’s alpha reliability of 0.69. A cut-off point for the SDSCA was not available in the body of literature in order to prepare it for a binary outcome analysis. Therefore, in this study participants who scored equal to or above the mean in the SDSCA were classified as having good diabetes self-care practice and those who scored below the mean were considered as having poor self-care practice. The *Revised Brief Diabetes Knowledge Test (DKT2)* [39] was used to measure diabetes knowledge. The DKT2 contains two sections: 14 general knowledge items appropriate for adults with Type 1 and Type 2 diabetes and 9 insulin use items appropriate only for adults with Type 1 diabetes. In this study, we have used only the general knowledge items. We have also dropped 5 of the general knowledge items since they were not meaningful for the cultural context of the study area. This was decided after consultation with nurses and physicians work in the diabetes clinics in the study area. Thus, 9 of the 14 general knowledge items were used in our study. In this study, participants who scored equal to or above the mean in the DKT2 were classified as having better knowledge of diabetes and those who scored below the mean were considered as having less knowledge. The *Diabetes Empowerment Scale – Short Form (DES-SF)* [40] was used to measure self-efficacy for diabetes self-care. The DES-SF is an 8-item scale and scored by averaging the scores of all completed items that have response option ranging from Strongly Disagree =1 to Strongly Agree = 5. It had coefficient alpha reliability of 0.84 in previous study [40]. In this study, participants who score equal to or above the mean in the DES-SF were classified as having better self-efficacy for diabetes self-care and those who scored below the mean were considered as having low self-efficacy. The *Brief Scale for Social Support (CAS)* [41] was used to measure social support. The CAS is a multidimensional 9-item scale with 5-point response options that range from none = 1 to very much = 5. The scale had internal consistence coefficient of 0.68 for the seven need items and 0.89 for two satisfaction items in previous study [41]. In this study, participants who scored equal to or above the mean in the CAS were classified as having better social support for self-care and those who scored below the mean were considered as having less social support.

### Data processing and analysis

Data was entered and cleaned using Epi Info version 3.5 and then transported to SPSS for Windows version 21.0 for analysis. Frequency distributions were computed for demographic variables and mean values with standard deviations were calculated for both the individual dimensions SDSCA and overall self-care practice, diabetes knowledge, self-efficacy, and social support scales. Pearson’s correlation was computed to explore the bivariate correlation between diabetes self-care, diabetes knowledge, self-efficacy, and social support. Then participants who obtained above the mean in overall self-care score were categorized as having good self-care practice and those who obtained below mean score as having poor self-care practice. We used the mean to dichotomize the self-care score because there was no cut-off point suggested in the body of literature. The proportion of participants with good and poor self-care practice was computed. We have used simple and multiple logistic regressions to analyze the association between: (1) demographic variables (age, gender, marital status, and education) and self-care practice, (2) clinical variable (time since diabetes diagnosis) and self-care practice, and (3) psychosocial variables (diabetes knowledge, self-efficacy, and social support) and self-care practice. We have used the dichotomized scores of diabetes knowledge, self-efficacy, and social support for the logistic regression analysis, with the dichotomization based on their mean values. The means were used to dichotomize these variables as there were no cut-off points suggested in the literature. A *p*-value below 0.05 and 95% confidence interval were used to evaluate the statistical significance of association between the predictor variables and diabetes self-care practice.

## Results

### Demographic and clinical characteristics

As shown in Table 1, from the total 257 participants in this study, 120 (46.7%) were in the age group of 40–59 years and the mean age was 42.9 ± 14.6 years. The majority of the participants were male (*n* = 139, 54.1%), Orthodox Christians (*n* = 139, 54.1%), and married (*n* = 173, 67.3%). Most of them lived with the disease for more than 10 years (*n* = 90, 35%) and the vast majority (*n* = 199, 77.4%) had no glucometer at home. Table 1 also shows a disaggregated mean score for the overall SDCSA and for the individual dimensions of SDCSA. Accordingly, blood glucose testing had the lowest self-care practice mean score across the range while foot care and medication taking had the highest mean scores.

**Table 1:** Demographic and clinical characteristics of study participants by SDCSA score (*n* = 257)

Variable	Number (%)	Overall SDCSA score, Mean (SD)	SDCSA Dimensions, Mean (SD)				
			Diet	Exercise	Glucose test	Foot care	Medication
Age in years:							
20–39	53 (20.6)	44.6 (7)	3.6 (1.1)	3.3 (2.2)	1.7 (0.9)	6.9 (0.5)	6.8 (0.8)
40–59	120 (46.7)	37.2 (9.4)	2.6 (1.5)	2.1 (1.9)	1.7 (1.2)	6.3 (1.3)	6.7 (0.9)
≥ 60	84 (32.7)	40.6 (9.6)	3.1 (1.5)	2.7 (1.9)	1.8 (1.1)	6.4 (1.1)	6.6 (1.2)
Sex:							
Male	139 (54.1)	42 (9.4)	3.2 (1.5)	2.9 (2.1)	1.9 (1.1)	6.5 (0.9)	6.7 (1.1)
Female	118 (45.1)	37.2 (8.9)	2.7 (1.5)	2 (1.7)	1.6 (1.1)	6.4 (1.2)	6.7 (0.9)
Religion:							
Orthodox Christian	139 (54.1)	38.4 (9.2)	2.7 (1.5)	2.4 (2.0)	1.7 (1.1)	6.4 (1.1)	6.6 (1.1)
Protestant Christian	91 (35.4)	41.2 (9.4)	3.2 (1.5)	2.7 (1.9)	1.7 (1.1)	6.5 (1.1)	6.7 (0.9)
Muslim	22 (8.6)	40.4 (10.3)	3 (1.5)	2.4 (2.3)	1.8 (1.5)	6.5 (1.0)	6.9 (0.5)
Waaqeffannaa	5 (1.9)	50.6 (1.1)	4.4 (0.5)	4.2 (0.8)	2.2 (0.4)	6.7 (0.4)	–
Educational level:							
Can’t read and write	39 (15.2)	40.6 (8.9)	3.1 (1.5)	2.6 (1.9)	1.7 (1.1)	6.4 (1.2)	6.7 (0.9)
Read and write	17 (6.6)	41.5 (7.4)	2.9 (1.2)	3.1 (2.7)	1.7 (0.9)	6.8 (0.8)	6.7 (0.9)
Primary school	90 (35%)	40.3 (7.9)	3 (1.4)	2.6 (1.9)	1.7 (1.0)	6.6 (0.9)	6.6 (1.0)
Secondary school	49 (19.1)	38.1 (9.4)	2.9 (1.5)	1.7 (1.6)	1.8 (1.1)	6.5 (0.9)	6.7 (0.9)
College/above	62 (24.1)	39.7 (12)	2.8 (1.7)	2.9 (2.2)	1.7 (1.2)	6.2 (1.4)	6.8 (1.1)
Marital status:							
Single	59 (23)	42.1 (9.2)	3.3 (1.4)	2.9 (2.1)	1.7 (0.9)	6.5 (1.1)	6.8 (0.7)
Married	173 (67.3)	38.9 (9.5)	2.8 (1.5)	2.4 (2.1)	1.7 (1.1)	6.4 (1.1)	6.7 (1.1)
Divorced	8 (3.1)	42.4 (7.2)	3.5 (1.6)	2.6 (1.2)	1.9 (0.9)	6.4 (0.7)	6.4 (1.2)
Widowed	17 (6.6)	40.5 (9.3)	3.1 (1.7)	2.4 (1.3)	2.3 (1.4)	6 (1.4)	6.7 (0.8)
Time since diabetes diagnosis:							
< 5 years	82 (31.9)	39.6 (9.8)	3 (1.5)	2.4 (1.9)	1.7 (1.1)	6.4 (1.2)	6.7 (0.9)
5–10 years	85 (33.1)	38.9 (9)	2.8 (1.5)	2.3 (1.9)	1.6 (1.0)	6.5 (0.9)	6.6 (1.1)
> 10 years	90 (35.0)	40.9 (9.6)	3 (1.5)	2.8 (2.2)	1.8 (1.2)	6.4 (1.1)	6.7 (0.9)
Family history of diabetes:							
Yes	47 (18.3)	42.8 (11.5)	3.1 (1.6)	3.5 (2.5)	1.7 (1.3)	6.7 (0.9)	6.5 (1.3)
No	163 (63.4)	39.1 (9.5)	2.9 (1.5)	2.3 (1.9)	1.8 (1.1)	6.4 (1.1)	6.8 (0.9)
I don’t know	47 (18.3)	39.3 (6.1)	3.1 (1.3)	2.3 (1.4)	1.6 (1.0)	6.4 (1.1)	6.6 (0.9)
Have glucometer at home:							
Yes	58 (22.6)	37.5 (10.6)	2.8 (1.6)	1.7 (1.7)	1.5 (0.9)	6.2 (1.4)	6.6 (1.2)
No	199 (77.4)	40.5 (9)	3 (1.5)	2.6 (2.1)	1.8 (1.1)	6.5 (1.0)	6.7 (0.9)

### Diabetes self-care practice and correlations

The overall mean score of diabetes self-care was 39.8 ± 9.5. Of the total participants, 45.5% score below the mean on the SDCSA measure, indicating poor self-care practice. Participants also had mean score of 5.6 ± 1.2 on diabetes knowledge scale (DKT2), 26.9 ± 4.3 on self-efficacy scale (DES-SF), and 27.1 ± 4.2 on social support scale (CAS). In this regard, most of the study participants scored above the mean on diabetes knowledge scale (53.3%), self-efficacy scale (54.1%), and social support scale (62.6%).

Table 2 depicts the correlation between individual SDCSA dimensions and DKT2, DES-SF, and CAS. Accordingly, dietary self-care practice was negatively correlated with DKT2, DES-SF, and CAS (*p*-value < 0.01). Exercise was also negatively correlated with DES-SF (*p*-value < 0.01), and with DKT2 and CAS (*p*-value < 0.05). Both foot care and medication taking were negatively correlated with DES-SF and CAS (*p*-value < 0.01) but not with DKT2. However, blood glucose testing was negatively correlated only with DES-SF (*p*-value < 0.01). Nevertheless, there were strong positive correlations among DKT2, DES-SF, and CAS (*p*-value < 0.01).

**Table 2:** Mean scores and correlation coefficient of self-care dimensions, diabetes knowledge, self-efficacy, and social support (*n* = 257)

Variable		Mean (SD)	1	2	3	4	5	6	7	8
1.	Diet	2.9 (1.5)	1							
2.	Exercise	2.5 (2.0)	0.25**	1						
3.	Glucose testing	1.7 (1.1)	0.12	0.02	1					
4.	Foot care	6.4 (1.1)	0.43**	0.03	0.08	1				
5.	Medication	6.7 (0.9)	0.09	0.07	0.02	0.15*	1			
6.	Diabetes knowledge	5.6 (1.2)	−0.29**	−0.16*	−0.12	−0.06	−0.04	1		
7.	Self-efficacy	26.9 (4.3)	−0.80**	−0.56**	−0.29**	−0.54**	−0.17**	0.29**	1	
8	Social support	27.1 (4.2)	−0.35**	−0.15*	−0.03	−0.32**	−0.16**	0.18**	0.41**	1

*p-value < 0.05 (2-tailed); **p-value < 0.01 (2-tailed)

### Predictors of self-care practice

In order to explore factors that have association with self-care practice both simple and multiple logistic regression analysis were used. Variables that had association with self-care practice at significant level (*p*-value) less than 0.2 in the simple logistic regression analysis were taken as candidates for multiple logistic regression analysis. These include age, marital status, education level, time since diagnosis, diabetes knowledge, and self-efficacy and social support (Table 3). In the simple logistic regression analysis, participants in the age group of 40 to 59 years old had 6.35 times higher self-care practice compared to those who were 20 to 39 years old (COR = 6.35, 95% CI = 3.11, 12.96). However, this did not hold in the multiple regression logistic analysis. Married participants had 3.4 times higher self-care practice compared to single participants in the simple logistic regression analysis (COR = 3.40, 95% CI = 1.82, 6.34). Again this faded out in the multiple logistic regression analysis. Compared to participants who did not read and write those who attended secondary school had 3.37 times (COR = 3.37, 95% CI = 1.42, 8.01) and those who attended above secondary school had 3.30 (COR = 3.30, 95% CI = 1.44, 7.52) higher self-care practice in the simple logistic regression analysis. But, in the multiple logistic regression analysis attending secondary school only had association with self-care practice (AOR = 6.00, 95% CI = 1.90, 18.85). Compared to participants who lived with diabetes for less than 5 years those who lived with the disease for more than 10 years had 5.64 times higher self-care practice (COR = 2.91, 95% CI = 2.91, 10.93) in the simple logistic regression analysis and 5.55 times higher in the multiple logistic regression analysis (AOR = 5.55, 95% CI = 2.29, 13.44). Compared to participants who had less diabetes knowledge those who possessed better knowledge had 4.04 times higher self-care practice in simple logistic regression analysis (COR = 4.04, 95% CI = 2.40, 6.81) and 2.42 times higher in multiple logistic regression analysis (AOR = 95% CI = 1.22, 4.80). Participants with better self-efficacy had 3.75 times higher self-care practice compared to those who possessed low self-efficacy in simple logistic regression (COR = 3.75, 95% CI = 2.23, 6.30) and 3.3 times higher in multiple logistic regression analysis (AOR = 3.30, 95% CI = 1.64, 6.62). Similarly, compared to participants who had less social support those who got better social support had 3.78 times higher self-care practice in simple logistic regression analysis (COR = 3.78, 95% CI = 2.21, 6.44) and 2.86 time higher in multiple logistic regression analysis (AOR = 2.86, 95% CI = 1.37, 5.96).

**Table 3:** Predictors of diabetes self-care practice (*n* = 257)

Variables	Self-care practice		Crude Odds Ratio (COR, 95%CI)	Adjusted Odds Ratio (AOR, 95%CI)
	Poor	Good		
Age				
20–39	37	16	1	1
40–59	32	88	6.35 (3.11,12.96)*	0.73 (0.15,3.44)
= > 60	48	63	1.73 (0.83,3.59)	0.27 (0.05,1.41)
Marital status				
Single	39	20	1	1
Married	63	110	3.40 (1.82,6.34)*	3.25 (0.78,13.45)
Divorced	3	5	3.25 (0.70,15.00)	2.6 (0.23,29.18)
Widowed	12	5	0.81 (0.25,2.62)	0.54 (0.07,4.12)
Education				
Not read and write	24	16	1	1
Read and write	14	3	0.32 (0.07,1.30)	0.38 (0.63,2.35)
Primary school attended	43	41	1.43 (0.66,3.06)	1.39 (0.48,3.99)
Secondary school attended	16	36	3.37 (1.42,8.01)*	6.00 (1.90,18.85)*
Above secondary school	20	44	3.30 (1.44,7.52)*	2.53 (0.82,7.80)
Time since diabetes diagnosis				
< 5 years	53	29	1	1
5–10 years	42	43	1.87 (1.00,3.48)	1.41 (0.635,3.16)
> 10 years	22	68	5.64 (2.91,10.93)*	5.55 (2.29,13.44)*
Diabetes knowledge				
Have less knowledge	76	44	1	1
Have better Knowledge	41	96	4.04 (2.40,6.81)*	2.42 (1.22,4.80)*
Self-efficacy				
Have low self-efficacy	74	44	1	1
Have better self-efficacy	43	96	3.75 (2.23,6.30)*	3.30 (1.64,6.62)*
Social support				
Have less social support	63	33	1	1
Have better social support	54	107	3.78 (2.21,6.44)*	2.86 (1.37,5.96)*

*p-value < 0.05

## Discussion

Congruent with the postulate of the Theory of Diabetes Self-Care Management (TDSCM), [34] our study has shown that diabetes knowledge, self-efficacy, and social support are important correlates and predictors of self-care practice. These findings corroborate with that of other international studies indicating diabetes knowledge, self-efficacy, and social support as critical predictors of diabetes self-care practice [42–46]. The possession of higher diabetes-related knowledge and social support likely improve self-efficacy for self-care practice [25, 42]. However, studies show that diabetes-related knowledge could be affected by health literacy level [47, 48] though this was not addressed in our study. Health literacy, in short, refers to the capacity of individuals to obtain and use health-related information for health actions and decisions [49, 50]. This means that an individual with optimum health literacy can have the capacity for diabetes self-care practice and glycemic control through improved diabetes knowledge and self-efficacy.

Nevertheless, there is controversy concerning the path through which health literacy is linked to self-care behaviors and glycemic control. Some researchers reported diabetes knowledge as an important link [51] while others indicate social support [18]. Other researchers even reported self-efficacy as a direct link between health literacy and glycemic control without the mediation of self-care behaviors [52] whereas others have reported self-efficacy as link between health literacy and diabetes self-care [53]. The present study also indicates existence of negative correlation between diabetes self-care score and diabetes knowledge score, social support score and self-efficacy score whereas the last three variables are positively correlated. Though our study has clearly identified that these three variables as predictors of diabetes self-care practice, further study is warranted to clarify the path through which they are associated with diabetes self-care practice. Moreover, further study is needed to assess how health literacy influence diabetes knowledge and self-efficacy and linked with diabetes self-care practice and glycemic control.

This study also shows that participants with secondary school education and those who lived with diabetes for more than 10 years had good self-care practices. Studies from other settings also show that diabetes patients with higher level of educational [54, 55] and longer duration of diabetes [25] are engaged more in self-care practice. Higher level of education and longer duration of diabetes may contribute to improved diabetes knowledge [56, 57] which is in turn linked with enhanced self-efficacy. Particularly, long duration of diabetes may provide the patient with an opportunity for learning from day-to-day life experiences in addition to the teaching provided by health professionals during follow up visits. This imply that patients with low level of education and short duration of diabetes need to be given special emphasis as they may the necessary knowledge about the disease and efficacy for self-care practice.

This study revealed that participants in this study had modest self-care practice (mean = 39.8 ± 9.5) though the proportion of those who scored above the mean in the overall SDSCA was 54.5% which is a bit higher than the findings reported by previous studies from Ethiopia [29, 30] and Kenya [58]. This variation could be attributed to the difference in measurement tools used and operationalization of self-care practice by these studies. This study also indicate that majority of the participants (77.4%) had no access to glucometer which might have contributed to the identified low practice of blood glucose self-monitoring. Moreover, our study revealed that blood glucose testing had strong positive correlation with self-efficacy. This implies that finding a way to increase patients’ access to glucometer and implementing self-efficacy enhancement interventions may help to improve blood glucose testing.

### Strength and limitation of the study

There are a couple of strengths of the present study. First, it is the first study to investigate the association between diabetes knowledge, social support, self-efficacy, and diabetes self-care practice in Ethiopia. Second, the use of cross-sectional design provided sufficiently large sample size. Despite these strengths, this study has a number of limitations. Cross-sectional studies don’t provide the researcher with the ability to infer causality. The use of self-report measures for data collection can be a subject to recall bias that may affect measurement precision. The measurement tools used in this study (SDSCA, DKT2, DES-SF, and CAS) did not pass through a rigorous validation process and psychometric analysis except simple forward and backward translation from English to local languages by multilingual translators. A rigorous validation process and psychometric analysis is warranted for future research. The use of mean score to dichotomize the score obtained by these instruments can be a limitation though we were forced to do so by the absence of suggested cut-off points. Moreover, we did not measure HbA1c due to inadequate financial support to cover the cost. It was also not available in the patients’ clinical record. Therefore, this can be considered as a limitation of the study.

## Conclusions

In conclusion, this study shows that the participants had modest diabetes self-care practice. The findings indicate that diabetes knowledge, social support, self-efficacy, attainment of secondary education, and longer duration of diabetes are important predictors of good diabetes self-care practice. Thus, interventions involving education about diabetes, strengthening support system, empowerment for self-care are warranted. Further research is also warranted to clarify the role of health literacy in affecting diabetes knowledge and self-efficacy and how it is linked with diabetes self-care behavior and glycemic control.

## Abbreviations

AIDS: Acquired immune deficiency syndrome
AOR: Adjusted odds ratio
CAS: Brief Scale for Social Support
CI: Confidence interval
COR: Crude odds ratio
DES-SF: Diabetes Empowerment Scale–Short Form
DKT2: Revised Brief Diabetes Knowledge Test
DM: Diabetes
HIV: Human immunodeficiency virus
REC: Research and Ethics Committee
SD: Standard deviation
SDSCA: Summary of Diabetes Self-Care Activities
SPSS: Statistical Package for the Social Sciences
TDSCM: Theory of Diabetes Self-Care Management
